# Senescent dermal fibroblasts negatively influence fibroblast extracellular matrix‐related gene expression partly via secretion of complement factor D

**DOI:** 10.1002/biof.1512

**Published:** 2019-04-26

**Authors:** Tomonobu Ezure, Misato Sugahara, Satoshi Amano

**Affiliations:** ^1^ Global Innovation Center, Shiseido Yokohama Japan

**Keywords:** CFD, fibroblast, MMP1, SASP, senescence

## Abstract

Aging is associated with a decrease of extracellular matrix and an increase of senescent cells in the dermal layer. Here, to examine whether and how senescent cells are involved in aging‐related deterioration of the dermal layer, we cocultured dermal young fibroblasts (low‐passage number) with senescent cells (high‐passage number) in Transwells, in which the two cell types are separated by a semipermeable membrane. Young fibroblasts in coculture showed decreased collagen type I alpha 1 chain and elastin gene expression, and increased matrix metalloproteinase 1 (MMP1) gene expression. To identify causative factors, we compared gene expression of young and senescent cells and selected candidate secretory factors whose expression was increased by ≥2.5 in senescent fibroblasts. Then, we used siRNAs to knock down each of the 11 candidate genes in senescent fibroblasts in the coculture system. Knockdown of complement factor D (CFD) in senescent fibroblasts significantly reduced the increase of MMP1 in the cocultured young fibroblasts. In monocultures, treatment of young fibroblasts with CFD resulted in increased MMP1 gene expression, while knockdown of CFD in senescent fibroblasts decreased MMP1 gene expression. In addition, production of CFD was increased in culture medium of untreated senescent fibroblasts. Furthermore, CFD gene and protein expression were increased in the dermal layer of skin specimens from aged subjects (>70 years old), compared to young subjects (<20 years old). Overall, these results suggest that senescent cells negatively influence matrix production and promote degradation of nearby fibroblasts in the dermal layer, in part through secretion of CFD.

**Abbreviations**
BAD: BCL2 associated agonist of cell death
CFD: complement factor D
COL1A1: collagen type I alpha 1 chain
MMP1: matrix metalloproteinase 1
PDL: population doubling level
PRAS40: proline‐rich Akt substrate of 40 kDa
SASP: senescence‐associated secretory phenotype

## 1 INTRODUCTION

Cellular senescence is associated with reduced cell proliferation, and has a critical influence on the response to cellular damage, inhibiting the progression of aberrant cells.^1^, ^2^ Further, senescent cells contribute to age‐associated tissue disorders, such as impaired tissue homeostasis and tumorigenesis, because of the impairment of tissue regeneration.^3^, ^4^ Senescent cells have been reported to secrete various cytokines (the so‐called senescence‐associated secretory phenotype [SASP]), that influence the tissue microenvironment and disrupt tissue structure and function through a paracrine effect.^5^ Indeed, removal of senescent cells has been reported to improve aging‐associated disorders in a mouse model.^6^

The presence of an aging‐induced factor was demonstrated by means of parabiosis experiments.^7^, ^8^ When old mice were surgically linked to young mice so that their blood mutually circulated, some tissues of the young mice developed aging‐associated phenotypes, such as delay of regeneration after injury. This effect was mediated by complement factor C1q.^9^ On the other hand, complement factor D (CFD) is expressed in various tissues, and has a role in the alternative pathway of complement activation.^10^ CFD, which is also known as adipsin, is present in adipose tissue, and may be related to energy homeostasis.^11^ But, it is not clear whether CFD production is related to aging.

The dermal layer contains abundant extracellular matrix, composed of collagen and elastin, which contribute to skin elasticity and serve to protect the inner tissue and retain the superficial morphology. With aging, the amount of matrix decreases,^12^ leading to loss of skin elasticity, delay of wound healing,^13^ skin ulcer formation,^14^ and changes of superficial morphology.^15^ Also, senescent cells have been reported to increase with aging in the dermal layer.^16^ However, it is still not clear whether and how senescent cells and SASP contribute to skin aging.

herefore, in order to clarify the role of secretory factors released from senescent fibroblasts in the dermal layer, we investigated the effects of senescent dermal fibroblasts on young dermal fibroblasts by using a coculture system in which the two types of cells are separated by a semipermeable membrane. We also aimed to identify the factor(s) mediating these effects.

## 2 EXPERIMENTAL PROCEDURES

### 2.1 Materials

Adult human (age 21) dermal fibroblasts were established from back skin as previously reported.^17^ Neonatal fibroblasts were purchased from Cascade Biologicals (Portland, OR) as a reference. Fetal bovine serum (FBS) was purchased from Sigma (St. Louis, MO). Superscript VILO, Lipofectamine and Dulbecco's modified Eagle's medium (DMEM) were from GIBCO/BRL (Carlsbad, CA). Cell culture flasks, dishes and Transwells were from Falcon (Franklin Lakes, NJ). siRNA for CFD (SI00030100 or SI00030107; similar inhibitory effects) and negative control siRNA (SI03650318), other siRNAs, RNeasy Protect Kit and Qiazole were purchased from Qiagen (Valencia, CA). CFD was from Life Technologies (Carlsbad, CA). Rabbit anti‐CFD antibody (LS‐C168701) was purchased from LifeSpan BioSciences (Seattle, WA). The Envision system was purchased from Dako (Carpenteria, CA). HRP‐linked anti‐rabbit IgG antibody was purchased from GE Healthcare (Braunschweig, Germany). PathScan® Antibody Array and rabbit anti‐phospho‐AKT antibody (4060) were purchased from Cell Signaling Technology (Boston, MA). Micro BCA™ Protein Assay kit was purchased from Thermo Fisher Scientific (Fair Lawn, NJ).

### 2.2 Cell culture system

Fibroblasts were cultured in DMEM containing 10% (w/v) FBS, in a humidified atmosphere of 5% CO_2_ in air at 37°C, and plated on a 6‐well Transwell system (Falcon, Franklin Lakes, NJ) at a density of 1,250 cells per cm^2^ (young cells: upper layer). Subconfluent senescent fibroblasts and young fibroblasts were seeded into the two compartments of the Transwell (senescent cells: lower compartment). Two days later, fibroblasts in the upper or lower compartment were harvested with Qiazole. Senescent cells were transfected with siRNA by means of Lipofectamine according to the instruction manual. UVB‐irradiated fibroblasts received a dose of 25 mJ.^18^ Further, the culture medium was collected and centrifuged, and the CFD concentration was determined by ELISA (R&D Systems, MN, USA) according to the instruction manual. Western blotting was conducted according to the instruction manual of the Amersham ECL system. Each cell lysate were normalized by protein concentration, measured with a Micro BCA™ Protein Assay kit.

### 2.3 Quantitative real‐time PCR

RNA was extracted with a RNeasy Protect Kit and translated to cDNA using Superscript VILO according to the manufacturer's instructions. 28S rRNA was quantified as an internal control, and quantification of cDNA for each selected gene was conducted by real‐time PCR amplification using a LightCycler.^19^ The cycle threshold values (Ct values) calculated by Lightcycler software version 3.5 were normalized to GAPDH mRNA for each sample. Primers and probes used in this study are as follows. *MMP1* forward; ATTTGCCGACAGAGATGAAGTCC, reverse; GGGTATCCGTGTAGCACATTCTG, *COL1A1* forward; AGCAGGCAAACCTGGTGAAC, reverse; AACCTCTCTCGCCTCTTGCT, *ELN* forward; TGTCCATCCTCCACCCCTCT, reverse; CCAGGAACTCCACCAGGAAT, *GAPDH* forward; GAAGGTGAAGGTCGGAGT, reverse; GAAGATGGTGATGGGATTTC, *CFD* forward; CATCTGGTTGGTCTTTATTGAGC, reverse; CATGCTGATCTCGAACTCCTG, MMP14 forward; TCCATCAACACTGCCTACGA, reverse; CACCCAATGCTTGTCTCCTT.

### 2.4 Gene expression analysis of dermal layer

The ethics committee of Shiseido Research Center approved all studies. Informed consent was obtained from each subject prior to all studies. Skin specimens were excised from the inner side of an upper arm of 8 female subjects in their 20s and 70s. The specimens were fixed with OCT compound (Sakura, Tokyo, Japan), and frozen at −80°. Sections of 10 μm were stained with hematoxylin, and then the dermal layer was microdissected and collected in Qiazole for extraction of RNA.

### 2.5 Histochemistry

Skin specimens fixed with acetone were embedded in paraffin according to the AMeX procedure.^20^ Sections of 5 μm were deparaffinized, rehydrated through graded alcohols, and then subjected to staining with hematoxylin–eosin. Frozen skin specimens fixed with OCT compound were sectioned at 8 μm and immunohistochemically stained with anti‐CFD antibody with the Envision system.

### 2.6 Statistical analysis

All data were expressed as means ± *SEM*. Differences between groups were examined for statistical significance using Student's *t* test or Dunnett's test as a multiple comparison test. A *p*‐value of less than .05 was considered to indicate a significant difference.

## 3 RESULTS

### 3.1 Senescent dermal fibroblasts decrease collagen type I alpha 1 chain and elastin gene expression and increase matrix metalloproteinase 1 gene expression of cocultured young fibroblasts

**Figure 1:**
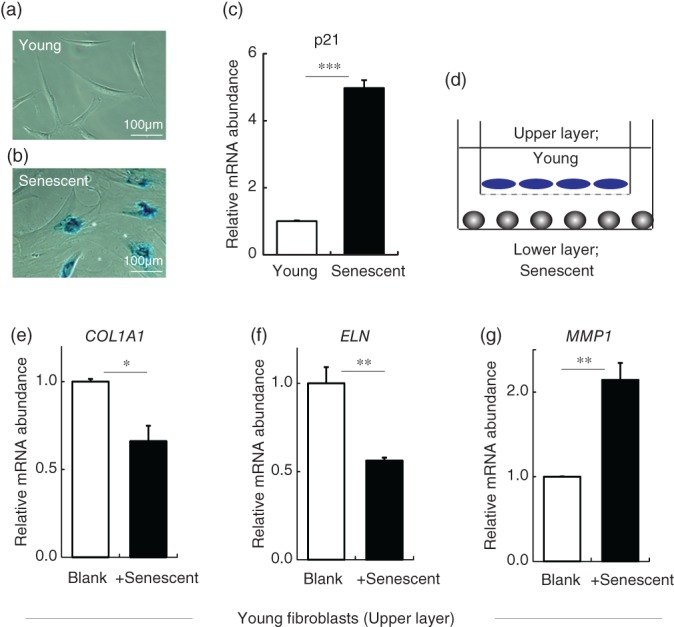
Senescent dermal fibroblasts increase MMP1 gene expression and decrease COL1A1 and elastin gene expression in young dermal fibroblasts. (a and b) Young fibroblasts were negative for beta‐galactosidase (a), whereas senescent fibroblasts were positive (b). (c) Senescent fibroblasts also showed increased p21 gene expression. (d) Coculture system of young dermal fibroblasts with (+senescent) or without (Blank) senescent fibroblasts. (e–g) Gene expression levels in young fibroblasts cultured alone (Blank) or cocultured with senescent dermal fibroblasts: (e) COL1A1, (f) elastin, and (g) MMP1. *p < .05, **p < .01 by Student's *t* test. Abbreviations: COL1A1, collagen type I alpha 1 chain; MMP1, matrix metalloproteinase 1

As senescent fibroblasts are reported to increase in the aged dermal layer,^16^ we constructed a coculture model in order to examine in detail the influence of senescent cells on young cells. Senescent cells were obtained by long‐term culture of adult and neonatal human dermal fibroblasts (more than 3 months; population doubling level [PDL] ≥ 50),^21^ and showed slow proliferation (doubling time < 0.5 per week), typical senescent cell phenotype of enlarged cell shape (Figure 1a,b), beta‐galactosidase positivity, and increased p21 gene expression (a senescent cell marker) (Figure 1c). Since these properties were similar in cells of both origins, we used fibroblasts from adult in the following experiments (neonatal fibroblasts were also used as a source for reference). When young fibroblasts (PDL ≤ 30) were cocultured in Transwells with senescent fibroblasts (about 50 ≤ PDL ≤ 60; Figure 1d), their gene expression of collagen type I alpha 1 chain (COL1A1) and elastin was significantly decreased, while that of matrix metalloproteinase 1 (MMP1) was significantly increased (Figure 1e–g). No significant change occurred when young fibroblasts were cocultured with other young fibroblasts (data not shown). Thus, senescent fibroblasts appeared to have a negative influence on fibroblast matrix‐related gene expression. Since the two types of cells were separated by a semipermeable membrane, through which cells could not pass, this action is likely to be mediated by a secretory factor. As senescent cells have been reported to secrete various cytokines (SASP), this phenotype is expected to be involved.

### 3.2 CFD as a mediator of the negative influence of senescent dermal fibroblasts on young fibroblasts

**Figure 2:**
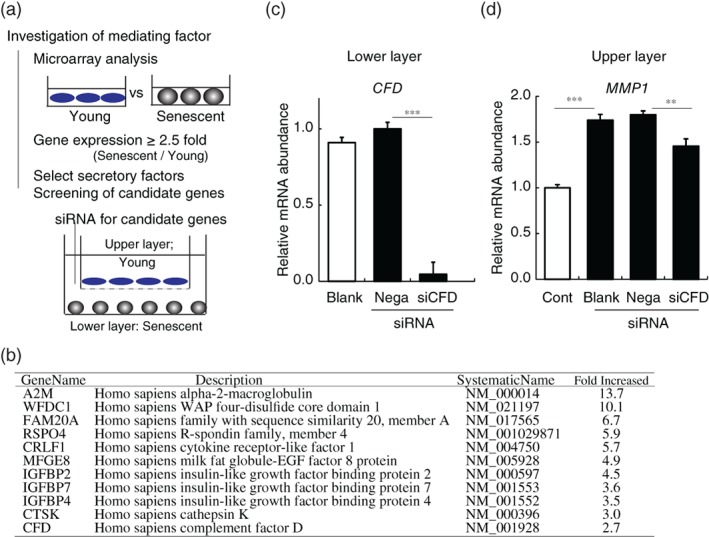
CFD mediates negative influence of senescent fibroblasts on young fibroblasts. (a) Strategy of screening to find mediators of the negative influence of senescent fibroblasts on young fibroblasts. Microarray analysis was conducted to find secretory factors (candidate genes) increased by 2.5‐fold or more in senescent fibroblasts. Candidate genes were knocked down in senescent fibroblasts (lower layer) by transfection with targeted siRNA (or control siRNA: Nega) in the coculture system, and changes of gene expression of the young fibroblasts (upper layer) were observed. (b) List of candidate genes (encoding secretory factors) whose expression was increased by 2.5‐fold or more in senescent dermal fibroblasts. (c) Gene expression of CFD in CFD‐knockdown senescent fibroblasts cocultured with young fibroblasts was almost completely blocked (siCFD), compared with the case of control siRNA‐transfected fibroblasts (Nega). (d) Gene expression of MMP1 in young fibroblasts cultured alone (Cont) or cocultured with senescent fibroblasts untreated (Blank) or transfected with siRNA directed at CFD (siCFD) or control siRNA (Nega). Knockdown of CFD reduced MMP1 expression, compared with Nega. **p < .01, ***p < .001 by Dunnett's test. Abbreviations: CFD, complement factor D; MMP1, matrix metalloproteinase 1

Next, we set out to identify the SASP secreted from senescent dermal fibroblasts. First, we conducted microarray analysis to compare gene expression in young and senescent fibroblasts (Figure 2a), and selected secretory factors that were increased by 2.5‐fold or more in the senescent fibroblasts (Figure 2b). Then, each of these candidate genes was knocked down in senescent fibroblasts, which were cocultured with young fibroblasts in the above system, and gene expression changes in the young fibroblasts were examined (data not shown). CFD‐directed siRNA almost completely blocked CFD mRNA expression in the senescent fibroblasts (Figure 2c). This knockdown of CFD in senescent fibroblasts significantly reduced the increment of MMP1 gene expression in cocultured young fibroblasts by about 50% (Figure 2d), while there was no significant change in COL1A1 or elastin gene expression in the young fibroblasts (data not shown). Knockdown of the other candidate genes had no effect on the negative influence of senescent fibroblasts on young fibroblasts in this coculture system. Thus, CFD appears to mediate the senescent fibroblast‐induced increase of MMP1 in young fibroblasts in the coculture system, at least in part.

### 3.3 Increase of CFD secretion by senescent fibroblasts negatively influences young fibroblasts

**Figure 3:**
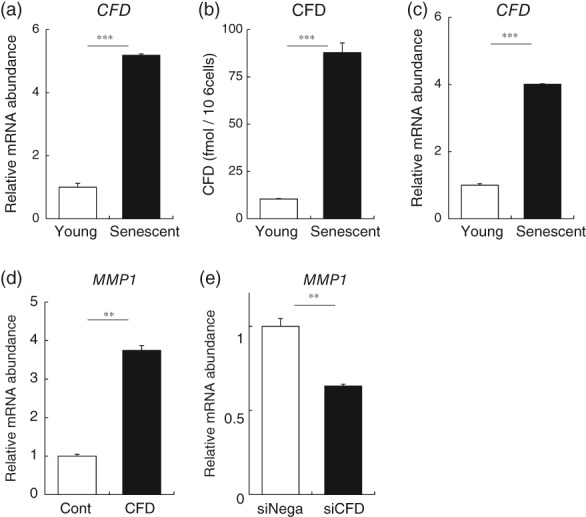
CFD is increased in senescent fibroblasts, and induces increased MMP1 expression in young dermal fibroblasts. (a) Gene expression of CFD was significantly increased in senescent fibroblasts. (b) CFD concentration in the culture medium of senescent fibroblasts was also significantly increased. (c) Gene expression of CFD was significantly increased in senescent fibroblasts derived from neonatal dermal fibroblasts. (d) Changes of MMP1 gene expression of CFD‐treated young fibroblasts. (e) Knockdown of CFD in senescent fibroblasts decreased MMP1 gene expression in the senescent fibroblasts. **p < .01, ***p < .001 by Student's *t* test. Abbreviations: CFD, complement factor D; MMP1, matrix metalloproteinase 1

Gene expression of CFD was significantly increased in senescent fibroblasts (Figure 3a), and we also confirmed the increase of CFD in the senescent fibroblasts derived from neonatal dermal fibroblasts from a different source as a reference (Figure 3c). CFD was also increased in UV irradiation‐exposed fibroblasts, which is another model of senescent fibroblasts induced by DNA damage^22^ (Figure S1). We further confirmed that CFD protein concentration in the culture medium of senescent fibroblasts was significantly increased (Figure 3b). To examine the effect of CFD, we added it to a culture of young dermal fibroblasts. We first confirmed that CFD was not cytotoxic by means of Alamar blue assay (Biosource, Camarillo, CA) (data not shown).^23^ We observed increased MMP1 gene expression in these fibroblasts (Figure 3d), in accordance with the change seen in the coculture system of senescent fibroblasts with young fibroblasts. In addition, CFD induced phosphorylation of AKT and AKT downstream molecules, that is, proline‐rich AKT substrate of 40 kDa (PRAS40) and BCL2 associated agonist of cell death (Bad), in young dermal fibroblasts as determined with a membrane array system followed by western blotting analysis (Figure S2a–e). Interestingly, CFD also increased MMP14 expression, which is regulated by the AKT pathway^24^ (Figure S2f). Addition of CFD did not induce cell damage, as judged from the absence of any change in apoptosis‐related factors, such as caspase 3, in membrane array assay. Intracellular AKT signaling is related to MMP1 induction in human dermal fibroblasts,^25^, ^26^ while the AP‐1 signaling pathway is involved in decrease of both COL1A1 and elastin.^27^, ^28^, ^29^ We also found that knockdown of CFD in senescent fibroblasts decreased MMP1 gene expression in the senescent fibroblasts themselves (Figure 3e). This was confirmed with another CFD siRNA having a different sequence. Overall, these results suggest that CFD could be a novel SASP secreted from senescent dermal fibroblasts.

### 3.4 CFD is increased in dermal layer of aged humans

**Figure 4:**
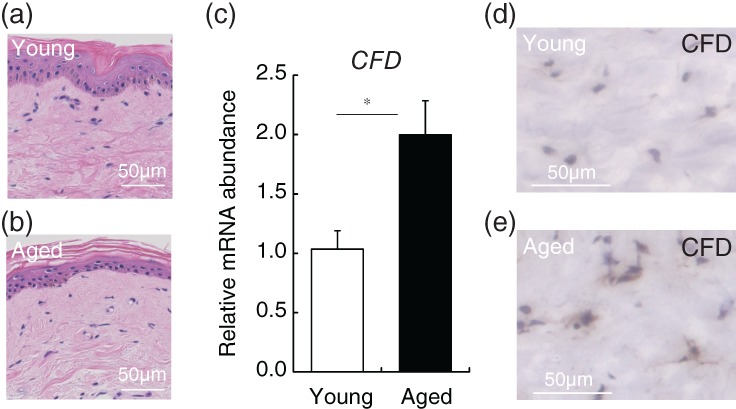
CFD is increased in aged human dermal layer. Skin specimens were biopsied from upper inner arm of eight young (in their 20s) and aged (in their 70s) subjects. HE‐stained dermal layer of (a) young and (b) aged subjects. (c) The dermal layer was laser‐capture microdissected, and gene expression analysis of CFD was conducted. *p < .05 by Student's *t* test. Immunohistochemistry of CFD in (d) young and (e) aged dermal layer. Abbreviations: CFD, complement factor D; HE, hematoxylin–eosin

Finally, we examined whether CFD is actually expressed in the dermal layer of aged subjects (in their 70s) and young subjects (in their 20s). Skin biopsies were taken from the upper inner arm (a sun‐unexposed area) and the dermal layer was collected with a laser‐capture microdissection system (Figure 4a–d). CFD gene expression in the dermal layer from aged subjects was significantly higher than in that from young subjects (Figure 4e). Immunohistochemistry confirmed that CFD was increased in the aged dermal layer (Figure 4f,g). As MMP1 is well established to increase in the aged dermal layer,^30^ these results support the idea that CFD could be a novel SASP in the dermal layer.

## 4 DISCUSSION

Our results indicate that senescent dermal fibroblasts negatively influence matrix‐related gene expression in nearby young dermal fibroblasts. We showed that CFD is secreted from senescent dermal fibroblasts and fibroblasts in an aged dermal layer, and we also found that CFD increased MMP1 gene expression in both senescent fibroblasts and young fibroblasts. Knockdown of CFD gene expression with siRNA significantly blocked the increase of MMP1 expression. Overall, our findings are consistent with the idea that CFD plays a role, at least in part, in mediating the age‐dependent changes of dermal matrix condition.

Senescent fibroblasts showed decreased COL1A1 and elastin gene expression, but inhibition of CFD or addition of CFD had no effect on expression of either gene. It is known that senescent fibroblasts secrete multiple factors,^31^ such as prostaglandins.^27^ In our coculture system, focusing on secretory proteins, we did not find any protein secreted by senescent fibroblasts that altered COL1A1 and elastin gene regulation, and further study would be needed to clarify the factors involved in suppression of the regulation of these genes. Nevertheless, our findings overall suggest that CFD is a senescence‐induced secretory factor that has a negative influence on surrounding fibroblasts.

Elastin and collagen type I are decreased in the aged dermal layer,^32^ due to both decreased synthesis and increased degradation.^33^ In this study, senescent cells decreased the levels of elastin and COL1A1 gene expression in surrounding fibroblasts, but as mentioned above, this effect was not mediated directly by CFD. However, CFD has been reported to possess elastase activity.^34^ Our present results also show that CFD induces MMP1, an enzyme that degrades collagen type I and is increased in fibroblasts in the aged dermal layer.^35^ Thus, senescent cells could contribute to the age‐dependent decrease of elastin and collagen type I through the action of CFD in terms of both degradation of elastin by its intrinsic elastase activity and induction of MMP1, leading to deterioration of the dermal matrix condition.

We found that CFD activated the AKT signaling pathway, and this could explain MMP1 induction by CFD, since Chung et al. reported that MMP1 is induced by AKT activation.^25^ However, the signaling pathways of AKT remain to be fully established, and further work will be needed to confirm this idea.

Targeting SASP could be an important strategy to control the negative influence of senescent cells. But, on the other hand, inhibition of SASP could allow proliferation of DNA‐damaged cells to occur, which might lead to tumor progression. Nevertheless, Demaria pointed out that, although MMPs secreted from senescent cells could chronically impair dermal condition, they contribute to the remodeling or hypertrophy of tissue during wound healing.^3^ Thus, SASP could have both positive and negative aspects. A better understanding of SASP and of its influence on intracellular signaling in target cells is needed to support the development of specific drugs for aging‐related disorders.

In conclusion, our results overall support the idea that senescent dermal fibroblasts, which are associated with aging, negatively influence surrounding fibroblasts via secretory factors. One such factor is CFD, which may be available as a target for control of aging‐related dermal disorders.

## CONFLICT OF INTEREST

The authors declare no conflict of interest.

## Supporting information


**Figure S1** Gene expression of CFD is increased in UV‐irradiated fibroblasts. Senescent fibroblasts induced by UVB showed significant increases of (a) a senescent cell marker (p21) and (b) CFD gene expression. **p < .01 by Student's *t* test

**Figure S2** CFD induces phosphorylation of AKT. (a–c) 30 min after addition of CFD to young fibroblasts, cell lysate was analyzed with a PathScan® Antibody Array. AKT phosphorylation was detected, together with phosphorylation of BAD and PRAS40, which are substrates of AKT. (d) List and location of signaling molecules in the membrane array. (e) AKT phosphorylation by CFD was confirmed by western blotting in terms of the cell lysate from 30 min after addition of CFD to the young fibroblasts (representative photograph from three experiments). (f) CFD induces MMP14 gene expression downstream of AKT activation. At 2 days after CFD addition to young fibroblasts, MMP14 expression was significantly increased. **p ≤ .01 by Student's *t* test

## References

[1] Campisi J . Cellular senescence as a tumor‐suppressor mechanism. Trends Cell Biol. 2001;11:S27–S31. 11684439 10.1016/s0962-8924(01)02151-1

[2] Wei W , Ji S . Cellular senescence: molecular mechanisms and pathogenicity. J Cell Physiol. 2018;233:9121–9135. 30078211 10.1002/jcp.26956

[3] Demaria M , Desprez PY , Campisi J , Velarde MC . Cell autonomous and non‐autonomous effects of senescent cells in the skin. J Invest Dermatol. 2015;135:1722–1726. 25855157 10.1038/jid.2015.108 PMC4466004

[4] Calcinotto A , Kohli J , Zagato E , Pellegrini L , Demaria M , Alimonti A . Cellular senescence: aging, cancer, and injury. Physiol Rev. 2019;99:1047–1078. 30648461 10.1152/physrev.00020.2018

[5] Gonzalez‐Meljem JM , Apps JR , Fraser HC , Martinez‐Barbera JP . Paracrine roles of cellular senescence in promoting tumourigenesis. Br J Cancer. 2018;118:1283–1288. 29670296 10.1038/s41416-018-0066-1 PMC5959857

[6] Baker DJ , Wijshake T , Tchkonia T , et al. Clearance of p16Ink4a‐positive senescent cells delays ageing‐associated disorders. Nature. 2011;479:232–236. 22048312 10.1038/nature10600 PMC3468323

[7] Brack AS , Conboy MJ , Roy S , et al. Increased Wnt signaling during aging alters muscle stem cell fate and increases fibrosis. Science. 2007;317:807–810. 17690295 10.1126/science.1144090

[8] Conboy MJ , Conboy IM , Rando TA . Heterochronic parabiosis: historical perspective and methodological considerations for studies of aging and longevity. Aging Cell. 2013;12:525–530. 23489470 10.1111/acel.12065 PMC4072458

[9] Naito AT , Sumida T , Nomura S , et al. Complement C1q activates canonical Wnt signaling and promotes aging‐related phenotypes. Cell. 2012;149:1298–1313. 22682250 10.1016/j.cell.2012.03.047 PMC3529917

[10] Xu Y , Ma M , Ippolito GC , Schroeder HW Jr , Carroll MC , Volanakis JE . Complement activation in factor D‐deficient mice. Proc Natl Acad Sci U S A. 2001;98:14577–14582. 11724962 10.1073/pnas.261428398 PMC64724

[11] Lo JC , Ljubicic S , Leibiger B , et al. Adipsin is an adipokine that improves beta cell function in diabetes. Cell. 2014;158:41–53. 24995977 10.1016/j.cell.2014.06.005 PMC4128197

[12] Xia W , Quan T , Hammerberg C , Voorhees JJ , Fisher GJ . A mouse model of skin aging: fragmentation of dermal collagen fibrils and reduced fibroblast spreading due to expression of human matrix metalloproteinase‐1. J Dermatol Sci. 2015;78:79–82. 25724359 10.1016/j.jdermsci.2015.01.009

[13] Knudsen AM , Gallagher S . Care of the obese patient with pressure ulcers. J Wound Ostomy Continence Nurs. 2003;30:111–118. 12658239 10.1067/mjw.2003.26

[14] Drake DJ , Swanson M , Baker G , et al. The association of BMI and Braden total score on the occurrence of pressure ulcers. J Wound Ostomy Continence Nurs. 2010;37:367–371. 20644369 10.1097/WON.0b013e3181e45774

[15] Ezure T , Hosoi J , Amano S , Tsuchiya T . Sagging of the cheek is related to skin elasticity, fat mass and mimetic muscle function. Skin Res Technol. 2009;15:299–305. 19624426 10.1111/j.1600-0846.2009.00364.x

[16] Lewis DA , Travers JB , Machado C , Somani AK , Spandau DF . Reversing the aging stromal phenotype prevents carcinoma initiation. Aging. 2011;3:407–416. 21515933 10.18632/aging.100318 PMC3117456

[17] Amano S , Ogura Y , Akutsu N , et al. Protective effect of matrix metalloproteinase inhibitors against epidermal basement membrane damage: skin equivalents partially mimic photoageing process. Br J Dermatol. 2005;153(Suppl 2):37–46. 10.1111/j.1365-2133.2005.06968.x 16280020

[18] Zhou BR , Guo XF , Zhang JA , et al. Elevated miR‐34c‐5p mediates dermal fibroblast senescence by ultraviolet irradiation. Int J Biol Sci. 2013;9:743–752. 23983607 10.7150/ijbs.5345 PMC3753410

[19] Ezure T , Amano S . Negative regulation of dermal fibroblasts by enlarged adipocytes through release of free fatty acids. J Invest Dermatol. 2011;131:2004–2009. 21697886 10.1038/jid.2011.145

[20] Sato Y , Mukai K , Watanabe S , Goto M , Shimosato Y . The AMeX method. A simplified technique of tissue processing and paraffin embedding with improved preservation of antigens for immunostaining. Am J Pathol. 1986;125:431–435. 2432790 PMC1888473

[21] Marasa BS , Srikantan S , Masuda K , et al. Increased MKK4 abundance with replicative senescence is linked to the joint reduction of multiple microRNAs. Sci Signal. 2009;2:ra69. 19861690 10.1126/scisignal.2000442 PMC2770878

[22] Greussing R , Hackl M , Charoentong P , et al. Identification of microRNA‐mRNA functional interactions in UVB‐induced senescence of human diploid fibroblasts. BMC Genomics. 2013;14:224. 23557329 10.1186/1471-2164-14-224 PMC4008267

[23] Page B , Page M , Noel C . A new fluorometric assay for cytotoxicity measurements in‐vitro. Int J Oncol. 1993;3:473–476. 21573387

[24] Sugg KB , Markworth JF , Disser NP , et al. Postnatal tendon growth and remodeling require platelet‐derived growth factor receptor signaling. Am J Physiol Cell Physiol. 2018;314:C389–C403. 29341790 10.1152/ajpcell.00258.2017 PMC5966786

[25] Oh JH , Kim A , Park JM , Kim SH , Chung AS . Ultraviolet B‐induced matrix metalloproteinase‐1 and ‐3 secretions are mediated via PTEN/Akt pathway in human dermal fibroblasts. J Cell Physiol. 2006;209:775–785. 16972255 10.1002/jcp.20754

[26] Hwang YP , Kim HG , Han EH , et al. N‐Acetylglucosamine suppress collagenases activation in ultraviolet B‐irradiated human dermal fibroblasts: involvement of calcium ions and mitogen‐activated protein kinases. J Dermatol Sci. 2011;63:93–103. 21600739 10.1016/j.jdermsci.2011.04.008

[27] Aaltomaa S , Lipponen P , Tammi R , et al. Strong stromal Hyaluronan expression is associated with PSA recurrence in local prostate cancer. Urol Int. 2002;69:266–272. 12444281 10.1159/000066123

[28] Jin YJ , Park I , Hong IK , et al. Fibronectin and vitronectin induce AP‐1‐mediated matrix metalloproteinase‐9 expression through integrin alpha(5)beta(1)/alpha(v)beta(3)‐dependent Akt, ERK and JNK signaling pathways in human umbilical vein endothelial cells. Cell Signal. 2011;23:125–134. 20816750 10.1016/j.cellsig.2010.08.012

[29] Xu X , Qin J , Liu W . Curcumin inhibits the invasion of thyroid cancer cells via down‐regulation of PI3K/Akt signaling pathway. Gene. 2014;546:226–232. 24910117 10.1016/j.gene.2014.06.006

[30] Fisher GJ , Quan T , Purohit T , et al. Collagen fragmentation promotes oxidative stress and elevates matrix metalloproteinase‐1 in fibroblasts in aged human skin. Am J Pathol. 2009;174:101–114. 19116368 10.2353/ajpath.2009.080599 PMC2631323

[31] Waldera Lupa DM , Kalfalah F , Safferling K , et al. Characterization of skin aging‐associated secreted proteins (SAASP) produced by dermal fibroblasts isolated from intrinsically aged human skin. J Invest Dermatol. 2015;135:1954–1968. 25815425 10.1038/jid.2015.120

[32] Sherratt MJ . Tissue elasticity and the ageing elastic fibre. Age. 2009;31:305–325. 19588272 10.1007/s11357-009-9103-6 PMC2813052

[33] Rossetti D , Kielmanowicz MG , Vigodman S , et al. A novel anti‐ageing mechanism for retinol: induction of dermal elastin synthesis and elastin fibre formation. Int J Cosmet Sci. 2011;33:62–69. 20704601 10.1111/j.1468-2494.2010.00588.x

[34] Zhu L , Wigle D , Hinek A , et al. The endogenous vascular elastase that governs development and progression of monocrotaline‐induced pulmonary hypertension in rats is a novel enzyme related to the serine proteinase adipsin. J Clin Invest. 1994;94:1163–1171. 8083356 10.1172/JCI117432 PMC295188

[35] Quan T , Little E , Quan H , Qin Z , Voorhees JJ , Fisher GJ . Elevated matrix metalloproteinases and collagen fragmentation in photodamaged human skin: impact of altered extracellular matrix microenvironment on dermal fibroblast function. J Invest Dermatol. 2013;133:1362–1366. 10.1038/jid.2012.509 PMC3637921 23466932

